# Brain Macro-Structural Alterations in Aging Rats: A Longitudinal Lifetime Approach

**DOI:** 10.3390/cells12030432

**Published:** 2023-01-28

**Authors:** Sidra Gull, Christian Gaser, Karl-Heinz Herrmann, Anja Urbach, Marcus Boehme, Samia Afzal, Jürgen R. Reichenbach, Otto W. Witte, Silvio Schmidt

**Affiliations:** 1 Department of Neurology, Jena University Hospital, Am Klinikum 1, D-07747 Jena, Germany; 2 Brain Imaging Center Jena, Jena University Hospital, Am Klinikum 1, D-07747 Jena, Germany; 3 Department of Psychiatry and Psychotherapy, Jena University Hospital, Philosophenweg 3, D-07743 Jena, Germany; 4 Medical Physics Group, Institute of Diagnostic and Interventional Radiology, Jena University Hospital, Philosophenweg 3, D-07743 Jena, Germany; 5 Jena Centre for Healthy Aging, Jena University Hospital, D-07747 Jena, Germany; 6 Biomagnetic Center, Department of Neurology, Jena University Hospital, Am Klinikum 1, D-07747 Jena, Germany

**Keywords:** rodent, animal, aging, brain, plasticity, volume, deformation-based morphometry, DBM, in vivo

## Abstract

Aging is accompanied by macro-structural alterations in the brain that may relate to age-associated cognitive decline. Animal studies could allow us to study this relationship, but so far it remains unclear whether their structural aging patterns correspond to those in humans. Therefore, by applying magnetic resonance imaging (MRI) and deformation-based morphometry (DBM), we longitudinally screened the brains of male RccHan:WIST rats for structural changes across their average lifespan. By combining dedicated region of interest (ROI) and voxel-wise approaches, we observed an increase in their global brain volume that was superimposed by divergent local morphologic alterations, with the largest aging effects in early and middle life. We detected a modality-dependent vulnerability to shrinkage across the visual, auditory, and somato-sensory cortical areas, whereas the piriform cortex showed partial resistance. Furthermore, shrinkage emerged in the amygdala, subiculum, and flocculus as well as in frontal, parietal, and motor cortical areas. Strikingly, we noticed the preservation of ectorhinal, entorhinal, retrosplenial, and cingulate cortical regions, which all represent higher-order brain areas and extraordinarily grew with increasing age. We think that the findings of this study will further advance aging research and may contribute to the establishment of interventional approaches to preserve cognitive health in advanced age.

## 1. Introduction

As life expectancy has increased in recent decades [1] and mortality among the elderly is predicted to decrease [2], the human population worldwide is growing older. Unfortunately, even in the absence of disease, advanced age is characterized by a cognitive decline that especially disturbs processing speed, working memory, and reasoning [3]. Nevertheless, a few aspects of cognition, such as semantic memory, the accumulation of knowledge, and the flexibility to switch between different executive tasks, are maintained or improved with increasing age [4,5]. Therefore, research into aging aims for the identification and manipulation of processes that drive such opposing cognitive trajectories to preserve cognitive health at an advanced age. In order to reach this goal, there is an urgent need for comprehensive information about functional and structural changes in the brain over the entire lifespan [6].

At the functional level, brain aging is characterized by a reorganization of distinct higher-order brain networks with reduced modular stability and de-differentiation of brain activation patterns during specific tasks [7]. Furthermore, there is an increased tendency of brain nodes to switch between different modules across time [8]. However, for technical reasons, functional measurements allow only task-specific statements, which, based on complex and unknown functional interactions, reflect only a fractionated picture of brain aging [4,9]. Nevertheless, due to the general relationship between function and structure, crucial changes that are functionally difficult to detect can be localized by using MRI-based morphometric methods [10,11,12,13,14,15,16,17]. 

At the structural level, it has long been known that the human brain suffers from differential atrophy and shrinks with increasing age [18,19,20,21,22]. Comprehensive integrative studies, covering the entire span of human life, have standardized the information about brain morphological changes and classified the cortex as especially vulnerable to age while some brain regions of hierarchically higher-order resist [23,24]. To explore the mechanisms driving such opposing morphological trajectories with the greatest methodological variety, experimental studies in rodents with defined laboratory conditions are appropriate [25]. In the present study, we screened the brains of male RccHan:WIST rats for aging-related macro-structural variability by using an MRI-based longitudinal approach. With a sampling rate of ten evenly distributed measurements including 3 to 30 months of age, this study provides the most comprehensive overview of brain volumetric changes in individual rodents available so far. Although the rodent brain grows with increasing age, we were able to expose a pattern of morphologic alterations that is consistent with differentiated cognitive decline and the spatial heterogeneity of aging in the human brain. 

## 2. Material and Methods

This article describes research on animals and does not contain any study with human participants. 

### 2.1. Animals and Experimental Design

Experiments were performed on male albino Wistar rats (RccHan:WIST; Envigo RMS B.V., 5961 NM Horst, Netherlands), housed in standard cages (2–5 animals per cage) on a 12 h light/dark cycle with ad libitum access to food and water. The hygiene status according to the Federation of European Laboratory Animal Science Associations (FELASA) was recorded regularly and documented by means of health certificates. All units were considered to be pathogen-free. The experimental protocols were approved by the ethical body of the Governmental Animal Care and Use Committee (Thüringer Landesamt für Lebensmittelsicherheit und Verbraucherschutz [TLLV], 99947 Bad Langensalza, Germany) and were performed in accordance with the guidelines and regulations of the European Commission on the protection of animals used for scientific purposes.

Ten longitudinal brain MRI scans covering most of the adult rat lifespan were acquired between 3 to 30 months of age at 3-month intervals. A total of n = 13 rats were included in the study resulting in a total of n = 130 scans. In addition to MRI, the body weight was measured immediately before the scans.

Since there are indications that anesthesia may have negative effects on brain physiology [26], we used the minimally noxious volatile agent isoflurane. Both dose and exposure time were kept at the lowest possible level. All animals included in the analyses were without phenotypic deviations and showed age-appropriate general and feeding behaviors, social interactions, and body conditions.

### 2.2. Magnetic Resonance Imaging (MRI)

As previously described by Herrmann et al. [27], MRI was performed on a clinical whole-body scanner (3T, Magnetom TIM Trio, Siemens Medical Solutions, Erlangen, Germany) by using a dedicated rat head volume resonator with a linearly polarized Litz design (Doty Scientific Inc., Columbia, SC, USA). Anesthesia of freely breathing animals was conducted using isoflurane (1.7% in oxygen, 1.5 L/min) and T2-weighted whole-brain images were obtained using a 3D SPACE sequence (Sampling Perfection with Application-Optimized Contrasts Using Different Flip Angle Evolutions, Siemens Healthcare, Erlangen Germany) with an isotropic resolution of 0.33 mm^3^ (matrix 192 × 130 × 96, FoV 64 × 43 × 32 mm^3^, bandwidth: 145 Hz/px, T_E_: 352 ms, T_R_: 2500 ms, flip angle mode: “T2var”, echo spacing: 10.7 ms, turbo factor: 67, Partial Fourier: 7/8 in both phase encode directions). The improved signal-to-noise ratio performance of the Doty coil enabled a protocol with two repetitions (on average) with TA = 14 min. 

### 2.3. Deformation-Based Morphometry (DBM)

Brain morphological changes were detected using the DBM tool established and described in detail by Gaser et al. [28]. The pipeline was implemented into the MATLAB software package SPM8 and works with a nonlinear registration approach. By these registrations, the brains were locally deformed (compressed or expanded) to match a reference brain. Thereby, morphologic differences between the image pairs were minimized and subsequently encoded in 3D deformation fields. The deformation-specific displacement vectors (Jacobian determinants) were then used to calculate the corresponding differences in volume at every voxel of the brain.

First, sequential images of animal-individual data sets were rigidly registered to their own baseline image from the 3-month time point. Then, local deformations were introduced by using a high-dimensional nonlinear registration step [29], which allowed the identification of subtle structural changes with great precision. The displacement vectors resulting from this step were used for whole-brain voxel-wise analyses. To assign each voxel to the coordinates of the Paxinos Atlas, in a second step, the intra-pair deformations were nonlinearly transformed onto a customized internal reference image (template brain) by using the default spatial normalization method implemented in SPM8. As this template brain was transformed into the space of the Paxinos Atlas [30], the parameters required for this step were used to calculate the total intracranial volume of individual brains as well as the volumes of selected atlas-based cortical regions of interest (ROIs; for the definition of cortical regions, see Supplementary Table S1B). Finally, the displacement vectors were smoothed with a Gaussian kernel with an FWHM of 0.6 mm and used for further statistical analyses.

### 2.4. Statistical Analyses

Temporal changes in intracranial brain volume and body weight were tested using a one-way repeated measures (RM) ANOVA with Tukey HSD. 

For the ROI-based analyses, the volume of distinct cortical regions was normalized to the respective intracranial brain volume and averaged across the hemispheres. Then, one-way RM ANOVA with Tukey HSD was used to test for changes over time in each region separately. Differences between selected sensory cortical regions (V1, AU1, S1, and Pir) were identified by using a two-way RM ANOVA with Tukey HSD. To check for differences between selected primary and secondary sensory cortical regions (V1 vs. V2, AU1 vs. Au2, and S1 vs. S2), the percentage volume changes were log10-transformed and tested using a two-way RM ANOVA with Tukey HSD. 

Whole-brain analyses for temporal brain morphological changes were performed voxel-wise within the MATLAB software package SPM8. A general linear model (GLM) was used with an RM ANOVA (threshold of *p* < 0.01, corrected for family wise error (FWE)). The analyses were adjusted for individual total intracranial volumes, and we tested for brain regions showing either a linear volume increase or decline. The results were color-coded and overlaid on the average of all Paxinos Atlas matched baseline images to visualize regions with a rate of volume change below (blue colors) or above (yellow colors) the rate of whole-brain volume change due to age. Finally, aging-related percentage volume changes from the four most significant local peaks in increasing or declining gray matter regions (averaged from the volume of a sphere with 0.6 mm in diameter) were plotted for graphical visualization, whereas one-way RM ANOVA with Tukey HSD was used on log10-transformed data to check for temporal changes over time in each region separately. 

## 3. Results

### 3.1. Body Weight

**Figure 1:**
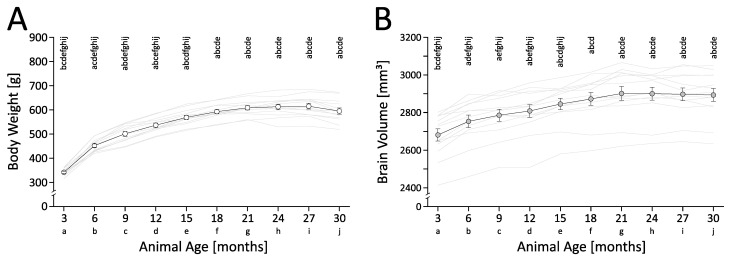
Bodyweight and total intracranial brain volume in male RccHan:WIST rats across lifetime. (**A**) Bodyweight increased successively until 18 months of age; the maximum was reached at 27 months of age. (**B**) Total intracranial brain volume increased between 3 and 6, and between 12 and 15 months of age; the maximum was reached at 21 months of age. Individual measures and the mean ± SEM are shown. A total of n = 13 rats were included in the study. The measurements, the estimated percentage changes and the statistics of one-way RM ANOVA with Tukey HSD are shown in Supplementary Table S1A. The results of the post hoc test are shown as age-coded letters, representing a significance level of *p* < 0.05.

In male RccHan:WIST rats, there was an asymptotic increase in body weight with increasing age (Figure 1A; F_(9,108)_: 313.27, *p* < 0.001, one-way RM ANOVA with Tukey HSD). The post hoc test indicated that successive body weight gains were significant up to the age of 18 months (*p* < 0.05). By a total gain of +79.94 ± 3.07%, animals reached their maximal body weight at 27 months of age (27 vs. 3 months, *p* < 0.05). The measurements, including estimated percentage changes as well as the statistics of the one-way RM ANOVA with Tukey HSD, are shown in Supplementary Table S1A.

### 3.2. Total Intracranial Brain Volume

Similar to body weight, the total intracranial brain volume increased in an asymptotic manner (Figure 1B; F _(9,108)_: 89.90, *p* < 0.001, one-way RM ANOVA, Tukey HSD). The post hoc test indicated that the brain grew significantly between 3 and 6 months of age (*p* < 0.05), as well as between 12 and 15 months of age (*p* < 0.05). With a total gain of +8.25 ± 0.59%, animals reached their maximal brain volume at 21 months of age (21 vs. 3 months, *p* < 0.05). Between 21 and 30 months there was no further growth, rather we observed a tendency toward a decline in brain volume. The measurements, including the estimated percentage changes as well as the statistics of the RM ANOVA with Tukey HSD, are shown in Supplementary Table S1A.

### 3.3. ROI-Based Analyses: Cortex

To examine how aging locally affects the brain structure, we individually normalized the age-specific regional estimates of 16 cortical regions to the respective total intracranial brain volume, averaged across the hemispheres, and analyzed the changes in these brain-size-corrected volumes longitudinally across time (for absolute measurements, brain-size-corrected volumes, and detailed statistics, see Supplementary Table S1C). This approach revealed cortical regions whose aging-related rate of volume change was below (henceforth termed “shrinkage”) or above (henceforth termed "growth") the rate of whole-brain volume change due to age [31]. 

**Figure 2:**
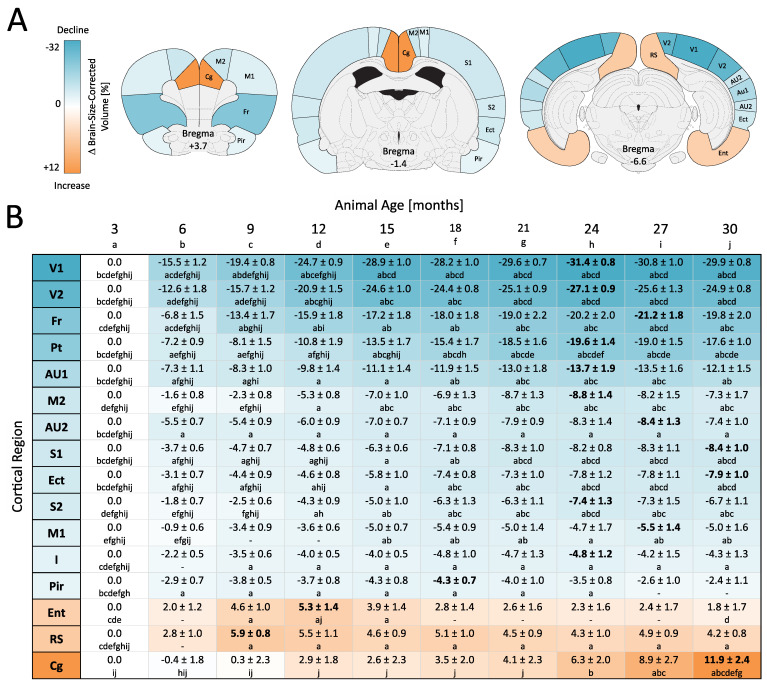
ROI-based analysis of cortical morphological changes in male RccHan:WIST rats across their lifetime. (**A**) The examined cortical regions are exemplarily superimposed on coronal sections from the Paxinos Atlas (V1/V2: primary/secondary visual, AU1/AU: primary/secondary auditory, S1/S2: primary/secondary somatosensory, Pir: piriform, M1/M2: primary/secondary motor, Fr: frontal, Pt: parietal, Ect: ectorhinal, I: insular, Ent: entorhinal, RS: retrosplenial, Cg: cingulate). The definitions of cortical regions are shown in Supplementary Table S1B. (**B**) Percentage changes in brain-size-corrected regional volumes are shown in ascending order as mean ± SEM (hemispheric averages, maxima in bold). Blue colors show regions for which the volume was changed below (henceforth termed “shrinkage”), and red colors show regions for which the volume was changed above (henceforth termed “growth”) the rate of whole brain volume change. A total of n = 13 rats were included in the study. The absolute measures, brain-size-corrected volumes, their estimated percentage changes, and the statistics of one-way RM ANOVA with Tukey HSD are shown in Supplementary Table S1C. The results of the post hoc test are shown as age-coded letters, representing a significance level of *p* < 0.05.

The majority of the investigated cortical areas (13 out of 16) were found to shrink (Figure 2). Consistently, trajectories followed a saturating progression, with the most pronounced shrinkage occurring between 3 and 15 months of age. However, there were differences in the extent of shrinkage. Ordered by descending effects, the maximum percentage changes in brain-size-corrected regional volumes (each vs. 3 months of age, mean ± SEM of bi-hemispheric average, one-way RM ANOVA with Tukey HSD) are as following: primary visual cortex (V1, −31.44 ± 0.81% at 24 months, F _(9,108)_: 182.00, *p* < 0.001), secondary visual cortex (V2, −27.09 ± 0.90% at 24 months, F _(9,108)_: 99.07, *p* < 0.001), frontal cortex (Fr, −21.18 ± 1.83% at 27 months, F _(9,108)_: 38.23, *p* < 0.001), parietal cortex (Pt, −19.62 ± 1.40% at 24 months, F _(9,108)_: 51.82, *p* < 0.001), primary auditory cortex (AU1, −13.67 ± 1.90% at 24 months, F _(9,108)_: 22.56, *p* < 0.001), secondary motor cortex (M2, −8.83 ± 1.44% at 24 months, F _(9,108)_: 15.70, *p* < 0.001), secondary auditory cortex (AU2, −8.36 ± 1.33% at 27 months, F _(9,108)_: 11.54, *p* < 0.001), primary somatosensory cortex (S1, −8.35 ± 0.97% at 30 months, F _(9,108)_: 22.22, *p* < 0.001), ectorhinal cortex (Ect, −7.91 ± 0.98% at 30 months, F _(9,108)_: 18.13, *p* < 0.001), secondary somatosensory cortex (S2, −7.42 ± 1.33% at 24 months, F _(9,108)_: 15.32, *p* < 0.001), primary motor cortex (M1, −5.47 ± 1.42% at 27 months, F _(9,108)_: 4.85, *p* < 0.001), insular cortex (I, −4.81 ± 1.22% at 24 months, F _(9,108)_: 4.56, *p* < 0.001) and piriform cortex (Pir, −4.33 ± 0.65% at 18 months, F _(9,108)_: 4.40, *p* < 0.001). 

Interestingly, the volume of different sensory areas was found to shrink in a modality-dependent manner (V1 > AU1 > S1 > Pir; F _(3,324)_: 156.10, *p* < 0.01 for all inter-areal comparisons—except for S1 vs. Pir with *p* = 0.09; two-way RM ANOVA with Tukey HSD). Further graduation was found within the visual and auditory areas, with the primary cortical areas shrinking more than the secondary ones (V1 > V2: F _(1,108)_: 57.64, *p*: <0.001; AU1 > AU2: F _(1,108)_: 7.45, *p* = 0.018; S1 > S2: not significant with F _(1,108)_: 1.65, *p* = 0.223; each two-way RM ANOVA, Tukey HSD)

In contrast to this widespread shrinkage, 3 out of the 16 investigated cortical regions increased in volume, each having a different trajectory and extent (Figure 2). As before, we estimated the maximum change in brain-size-corrected regional volumes for each region (each vs. 3 months of age, mean ± SEM of bi-hemispheric average, one-way RM ANOVA with Tukey HSD). This revealed that the volume of the entorhinal cortex initially grew up to 12 months of age (Ent; +5.33 ± 1.42% at 12 months; F _(9,108)_: 4.00, *p* < 0.001), whereupon volume gain diminished until 30 months of age. The retrosplenial cortex grew until 9 months of age (RS; +5.94 ± 0.77%; F _(9,108)_: 5.11, *p* < 0.001), showing no significant changes thereafter. The cingulate cortex showed the largest increase in volume with an almost linear progression until 30 months of age (Cg; +11.86 ± 2.35% at 30 months; F _(9,108)_: 7.61, *p* < 0.001).

### 3.4. Voxel-Wise Analyses: Whole Brain

In addition to the ROI-based analyses of selected cortical regions, we used a GLM to holistically screen the whole brain in a voxel-wise manner for aging-related morphologic variability. In analogy to the ROI-based analyses, the tests were corrected for total intracranial volume to reveal regions for which the volume increased less (henceforth termed “shrinkage”) or more (henceforth termed “growth”) than that of the whole brain [31]. 

**Figure 3:**
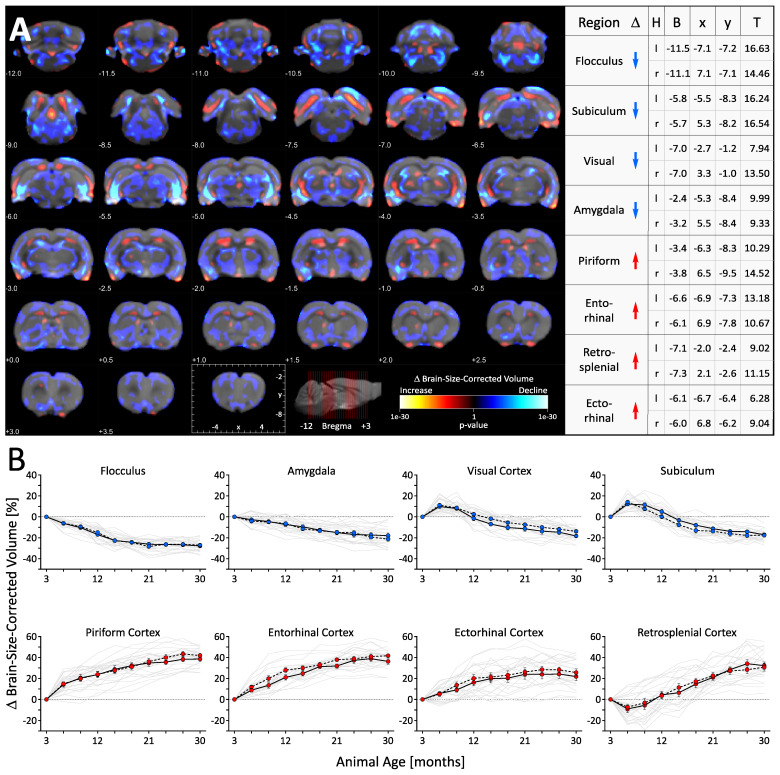
Voxel-wise analysis of whole brain morphological changes in male RccHan:WIST rats across their lifetime. (**A**) Statistical parametric maps demonstrating the spatial heterogeneity in aging-related macro-structural alterations. Results of the RM ANOVA analyzing temporal changes in brain-size-corrected local volume (threshold of *p* < 0.01, corrected for family wise error (FWE)) are superimposed on coronal sections of the reference brain. Blue colors show regions for which the volume was changed below (henceforth termed “shrinkage”), and red colors show regions for which the volume was changed above (henceforth termed “growth”) the rate of whole brain volume change. (**B**) Quantification of aging-related percentage volume changes in the four most significant shrinking and growing brain areas. Individual data and the average from the left (solid line) and right hemisphere (dotted line) are shown as mean ± SEM. A total of n = 13 rats were included in the study. Region-specific analyses for temporal changes using one-way RM ANOVA with Tukey HSD as well as the uncorrected changes are shown in Supplementary Table S1D. Abbreviations: H: hemisphere; l/r: left/right; B,x,y: Bregma and xy-coordinates corresponding to the Paxinos Atlas; T: T-value RM ANOVA, threshold of *p* < 0.01, corrected for family wise error (FWE).

Results exceeding the threshold of *p* < 0.01 (corrected for family wise error (FEW)) are shown in Figure 3A. The pattern of aging-related structural alterations proved to be heterogeneous and highly complex with expansive, ramified, and multiple interconnected clusters extending through the entire brain. The aggregated cluster sizes indicate that shrinking (blue colors, total cluster size: 24.188 voxels, T-value threshold > 5.5) clearly outreach regions with excessive growth (red colors, total cluster size: 3.261 voxels, T-value threshold > 5.5). We selected the four most significant focal peaks, each in shrinking and growing gray matter regions, for more detailed analyses. Their coordinates, the coordinates of the corresponding local maxima in the contralateral hemisphere, and the Paxinos Atlas-based allocation to specific brain areas are shown in Figure 3A. The respective percentage changes in volume were averaged across a 0.6 mm spherical volume around the peaks and plotted over time (Figure 3B). In analogy to the ROI-based analyses, we estimated the maximal percentage changes in brain-size-corrected volumes (each vs. 3 months of age, mean ± SEM of bi-hemispheric average, one-way RM ANOVA with Tukey HSD, for detailed statistics including respective measures based on analyses un-corrected for total brain volume, see Supplementary Table S1D). There was prominent local aging-related shrinkage in the flocculus (−27.41 ± 0.84% at 30 months, F _(9,108)_: 150.66, *p* < 0.001), the amygdala (−19.60 ± 1.89% at 30 months, F _(9,108)_: 42.28, *p* < 0.001), the subiculum (−17.36 ± 1.05% at 30 months, F _(9,108)_: 134.59, *p* < 0.001) and the visual cortex (−16.06 ± 1.13% at 30 months, F _(9,108)_: 73.98, *p* < 0.001). While the volume in the amygdala and the flocculus decreased continuously, shrinkage in the visual cortex and the subiculum started after initial growth (visual: +10.48 ± 1.27% at 6 months; subiculum: +13.11 ± 1.11% at 6 months). The most prominent local growth was observed in the piriform cortex (+40.87 ± 2.07% at 27 months, F _(9,108)_: 162.01, *p* < 0.001), the entorhinal cortex (+39.90 ± 2.00% at 27 months, F _(9,108)_: 181.29, *p* < 0.001), the retrosplenial cortex (+31.46 ± 2.56% at 30 months, F _(9,108)_: 70.88, *p* < 0.001) and the ectorhinal cortex (+26.23 ± 2.56% at 27 months, F _(9,108)_: 43.04, *p* < 0.001). While the volume in the piriform, ectorhinal and entorhinal cortex increased continuously, growth in the retrosplenial cortex started after initial shrinkage (−8.22 ± 2.31% at 6 months).

Overall, our MRI-based morphometric analyses indicate that the brains of male RccHan:WIST rats undergo variable and regionally opposing structural alterations, which mainly occur during young and middle age, whereupon the brain volume tends to stabilize at a higher age.

## 4. Discussion

Here, we longitudinally screened the brains of male RccHan:WIST rats for aging-related morphologic variability using an MRI-based approach. With a sampling rate of ten evenly distributed observation points including three to 30 months of age, this study provides the most comprehensive overview of brain volumetric changes in rodents available so far. At three different levels of spatial resolution (intracranial brain volume, ROI-based for selected cortical regions, and voxel-wise holistically), we reveal a heterogeneous and highly complex pattern of macro-structural alterations in the brain which predominantly occur during young and middle age and thereafter more or less stabilized at an advanced age.

First, we observed an increase in rat intracranial brain volume of approximately +8% between 3 and 21 months of age, which is consistent with results of previously reported MRI-based studies in rats [32,33,34,35] and mice [36,37,38,39,40]. Our longitudinal data add to this existing knowledge and show that global brain growth in male RccHan:WIST rats follow a biphasic saturating progression, with most changes occurring in young and middle age. This trajectory contrasts with the lifespan curve known from the human brain [22,41,42,43], which is characterized by a continuous volume loss starting in childhood, at approximately 13 years of age [23,24]. However, although we covered the average lifespan of Wistar rats, we cannot rule out a late shrinkage occurring at ages later than 30 months. A few animals of this strain have been reported to reach ages up to 50 months [44,45].

To examine how aging locally affects brain structure, we individually adjusted the age-specific regional estimates to the respective total intracranial volume and analyzed these brain-size-corrected volume changes longitudinally across time. This reveals regions for which the rate of volume change is below (henceforth termed “shrinkage”) or above (henceforth termed “growth”) the rate of whole-brain volume change due to age [31]. In line with previous reports from both rodents [39,40] and humans [20,23,24,31,46], we detected a pronounced heterogeneity within aging-related regional morphologic alterations that predominantly reflects shrinkage, while in rats a few regions show exceptional growth.

Our ROI-based approach revealed a modality-dependent decline in the volume of sensory cortical areas with graduated extent. While the largest effects of age were found in the visual, auditory, and somatosensory areas, the piriform cortex displayed the lowest shrinkage. Such a modality-dependent heterogeneity may reflect differential grades of degradation in the primary sensory input from the periphery, alongside disturbed central processing: a progressive loss of distal sensory cells and afferent inputs in the visual [47], auditory [48] and somatosensory system [49,50] is contrasted by the lifelong capacity for cell renewal in the olfactory epithelium, which preserves the amount of olfactory sensory neurons with stable receptor profiles and sensitivity into old age [51,52]. While the central mechanisms of olfactory adaptation and odor discrimination become largely preserved in aged rodents [53,54,55], the degradation of peripheral inputs in the somatosensory, auditory, and visual system accompanied by deteriorations in the topographic order of cutaneous receptive fields [56,57], temporal acoustic processing [58,59] and spatial visual perception [60,61]. Similar associations between modality-dependent sensory decline and local gray matter atrophy are known from the human brain as a consequence of retinal degeneration [62,63] or peripheral hearing loss [64,65,66,67]. Thus, our findings support the established concept that sensory degradation accelerates the aging of the brain and contributes to cognitive decline [68,69,70,71].

In contrast to this extensive shrinkage, which has a greater effect on the primary areas than the secondary areas of the sensory system, we detected the exceptional growth of the entorhinal, retrosplenial, and cingulate cortex, all of which represent areas of hierarchically higher order. The graduated preservation of brain areas towards a higher order was recently shown by Bethlehem et al. [24] in the human brain and may reflect various aspects of structure–function relationships [72]. For example, it has been shown that older animals benefit more from multimodal stimulus representations, whose processing is shifted from primary to higher sensory association areas [73]. Thus, while primary sensory areas suffer from peripheral input degradation, the functional relevance of less disconnected secondary sensory areas increases with age and potentially makes them less susceptible to degradation. Of particular significance seems to be the continuous enlargement of the cingulate cortex, which was recently shown by Fowler et al. [40] in mice. This area represents an evolutionarily conserved superior integration hub [74], that interconnects multiple but functionally distinct higher-order brain networks [75,76,77]. Its superior position enables the cingulate cortex to administrate a kind of metastability among these higher-order brain networks (e.g., default mode, fronto-parietal, dorsal attention, sensorimotor, and salience) and to coordinate the dynamic switches between different brain processing modes, which are crucial for efficient cognition and adequate reactions to physical and environmental demands [78]. Thus, regardless of the brain processing mode, this central area is under permanent challenge and by widely distributed feedback loops constantly accumulates diffuse aging-related changes from the entire brain. Since its structural preservation is related to superior fluid cognitive function in the aging human brain [79,80], it seems plausible that the cingulate cortex is subject to lifelong adaptive and function-preserving plasticity, which finally allows it to grow. This hypothesis is consistent with observations suggesting that the corrective adjustments and enduring availment of circumscribed neuronal networks over the course of the intensive exercise of various skills are associated with local gray matter growth [81,82,83,84,85,86,87]. In line with these considerations, the pronounced shrinkage of the frontal and parietal cortex could reflect aging-related imbalances in regional brain activity due to the deprived housing conditions of rats in our experimental design. Both areas are the main components of task-positive networks, which become activated during demanding and externally directed activities and are deprived during physically inactive periods [78,88]. As the exploratory activity of rats kept in standard cages decreases significantly with age [32], the shrinkage of frontal, parietal, and motor areas is consistent with their reduced use [89].

In addition to our ROI-based and cortex-focused analyses, the global holistic approach revealed the hotspots of aging-affected brain regions with higher spatial resolution. The complex pattern with expansive, ramified, and multiple interconnected clusters confirm the heterogeneity of structural changes and show that predefined atlas-based brain areas may age partially to different extents. Whereas the ROI-based analyses indicate that the piriform and ectorhinal cortices shrink as a whole, the holistic approach revealed circumscribed segments within them undergoing significant growth. Information about the function of the ectorhinal cortex is sparse, but it is thought to influence autonomic functions and the sympathetic nervous system [90,91]. However, the partial growth in the piriform cortex supports the above statements. As an indication of lifelong functional rodent olfaction, the associated cortex is largely preserved. The selective growth of the circumscribed segment could reflect olfactory specialization to the constant housing conditions, which in comparison to the natural habitat of the animals offers only a small fraction of sensory cues. Our holistic approach also highlights a volume decline in the flocculus, a cerebellar protrusion known as the adaptive control system of the vestibulo-ocular reflex, which synchronizes sensory perception with motor functions [92]. In addition to the degradation in the auditory and visual systems, the volume decline may reflect disturbed vestibular inputs [93] and associated postural, movement, and balance disorders, which significantly contribute to worsened general health conditions with increasing age in humans [94].

Additionally, in line with sensory input degradation, amygdala atrophy is one of the main structural correlates of cognitive decline in human brain aging [95,96], and their shrinkage is confirmed by our study in rats. The amygdala is crucial in the selection of relevant information from the constant sensory input stream, in linking this information with biological value, and in making decisions about the mobilization of additional brain and body resources to solve environmental demands [97].

The retrosplenial and entorhinal cortices represent strongly interconnected higher-order hub areas and considering functional aspects their structural growth with increasing age is not that surprising. Both the retrosplenial [98,99,100,101] and the entorhinal cortex [102,103] serve as long-term storage sites for navigational maps. However, most importantly, they represent the central storage sites for flexible associations between countless memory engrams distributed in the whole brain [104,105,106,107,108]. In particular, the storage of multimodal associations is a lifelong dynamic process based on sensory degradation and changing input statistics that change across the lifespan, requiring constant updates and restoration [109]. The exceptional growth is therefore compatible with the lifelong accumulation of dendritic spines, which are known to be crucial for memory consolidation [110,111,112,113]. Although not explicitly shown for the retrosplenial cortex, the entorhinal cortex is the most resistant to aging in humans [24,114]. In this context, our approach also emphasizes the shrinkage of the subiculum, which in humans represents the most aging-affected substructure of the hippocampus [115,116,117,118]. It plays a crucial role in the organization, processing, and distribution of the hippocampal output [119,120] and is especially important for memory retrieval [121,122,123,124] and the synthesis of retrieval-induced stress hormones that promote memory reconsolidation [125]. Therefore, together with structural preservation in entorhinal and retrosplenial cortices, the shrinkage of the subiculum supports the established concept that memory retrieval in particular becomes impaired with advancing age [126,127,128,129].

Our results conclusively suggest that established concepts for aging-related cognitive decline are locally reflected on the macro-structural level in the rodent brain. The complex pattern, including vulnerable and shrinking regions and those that demonstrate resistance and extraordinary growth, illustrates the spatial heterogeneity of brain aging processes, and accentuates disturbed sensory input and disproportional utilization as possible driving factors. We think that the findings of this study will further advance research on aging and may contribute to the establishment of interventional approaches to preserve cognitive health in advanced age. Therefore, rodents represent an appropriate model system to profoundly explore the mechanisms of brain aging on the cellular and molecular level in a reasonable frame of time—which is not yet possible to this extent in humans.

## Data Availability

The data presented in this study are available in the article or in the Supplementary Material. The raw MRI data as well as the MATLAB code used for the morphometric analyses are available from the corresponding author upon reasonable request.

## Supplementary Materials

The following supporting information can be downloaded at: https://www.mdpi.com/article/10.3390/cells12030432/s1, Table S1A: BW, BV; Table S1B: ROI Definition; Table S1C: ROI-based Cortex; Table S1D: Voxel-wise Whole Brain.
